# Evaluation of Risk Factors for Attention Deficit Hyperactivity Disorder in Sri Lankan Children: A school based population study from a developing nation

**DOI:** 10.1192/j.eurpsy.2022.598

**Published:** 2022-09-01

**Authors:** N. Nazeer, Y. Rohanachandra, S. Prathapan

**Affiliations:** 1 Ministry of Health, National Programme For Tuberculosis Control And Chest Diseases, Colombo, Sri Lanka; 2 University of Sri Jayewardenepura, Psychiatry, Colombo, Sri Lanka; 3 University of Sri Jayewardenepura, Community Medicine, Colombo, Sri Lanka

**Keywords:** Primary School Children, Sri Lanka, risk factors, adhd

## Abstract

**Introduction:**

Attention Deficit Hyperactivity Disorder (ADHD) is one of the most common psychiatric disorders of childhood with significant impairment in overall functioning and associated psychiatric comorbidities. Knowledge of determinants is vital for the development of effective preventive strategies and tailor made interventions.

**Objectives:**

The study was aimed at determining risk factors for the development of ADHD among primary school children (PSC)) in state schools in Colombo district of Sri Lanka.

**Methods:**

An unmatched case control study was conducted consisting of 73 cases (with ADHD) and 264 controls (without ADHD) selected randomly among 6-10 year old PSC from Sinhala medium state schools in Colombo district. Primary Care Givers completed the validated Sinhala version of Swanson, Nolan, Pelham –IV (SNAP-IV-S) scale for the assessment of ADHD and an interviewer administered questionnaire on risk factors. Children’s diagnostic status was confirmed by a Consultant Child and Adolescent Psychiatrist following a clinical assessment. Bivariate analysis followed by multivariate regression model identified potential risk factors.

**Results:**

Multivariate analysis revealed, male sex (aOR=3.74; 95%CI=1.67-8.35), lower educational level of the mother (aOR=3.31; 95%CI=1.39-7.98), maternal psycho pathology (aOR=7.28; 95%CI=1.55-34.35), prenatal exposure to passive tobacco smoke (aOR=3.76; 95%CI=1.09-12.95), Birth weight <2500g and /or gestation period of <37 completed weeks (aOR=3.6; 95%CI= 1.48-8.74), neonatal complications (aOR=4.03; 95%CI=1.94-8.32) , minimal leisure time with family (aOR=2.39; 95%CI=1.19-4.82) and subjected to teasing/ bullying (aOR=5.03; 95%CI=2.47-10.25) as significant predictors of ADHD.

**Conclusions:**

Primary prevention focusing on strengthening neonatal, maternal and child health services needs highlighting together with special emphasis on the need for anti-bullying policies in state schools.

**Disclosure:**

No significant relationships.

